# Social gradients in child and adolescent antisocial behavior: a systematic review protocol

**DOI:** 10.1186/2046-4053-1-38

**Published:** 2012-08-23

**Authors:** Patrycja J Piotrowska, Christopher B Stride, Richard Rowe

**Affiliations:** 1 Department of Psychology, University of Sheffield, Western Bank, Sheffield, S10 2TP, UK

**Keywords:** Antisocial behavior, Social gradient, Social inequalities, Conduct Disorder, Oppositional-Defiant Disorder, Socioeconomic status

## Abstract

**Background:**

The relationship between social position and physical health is well-established across a range of studies. The evidence base regarding social position and mental health is less well developed, particularly regarding the development of antisocial behavior. Some evidence demonstrates a social gradient in behavioral problems, with children from low-socioeconomic backgrounds experiencing more behavioral difficulties than children from high-socioeconomic families. Antisocial behavior is a heterogeneous concept that encompasses behaviors as diverse as physical fighting, vandalism, stealing, status violation and disobedience to adults. Whether all forms of antisocial behavior show identical social gradients is unclear from previous published research. The mechanisms underlying social gradients in antisocial behavior, such as neighborhood characteristics and family processes, have not been fully elucidated. This review will synthesize findings on the social gradient in antisocial behavior, considering variation across the range of antisocial behaviors and evidence regarding the mechanisms that might underlie the identified gradients.

**Methods:**

In this review, an extensive manual and electronic literature search will be conducted for papers published from 1960 to 2011. The review will include empirical and quantitative studies of children and adolescents (<=18 years old) recruited from the general population, which include measures of both social position and antisocial behavior. A standardized data extraction form and quality appraisal checklist will be used to retrieve essential information and critically appraise each study and the inter-rater reliability of the quality scores will be assessed. If practical, meta-analysis will be used to synthesize the data. However, it is expected that the selected studies will be heterogeneous, in which case narrative synthesis will be applied. Separate conclusions may be drawn for logically grouped studies on the basis of their quality score, scope or methodology.

**Discussion:**

This systematic review has been proposed in order to synthesize cross-disciplinary evidence of the social gradient in antisocial behavior and mechanisms underlying this effect. The results of the review will inform social policies aiming to reduce social inequalities and levels of antisocial behavior, and identify gaps in the present literature to guide further research.

## Background

Social inequalities have been extensively studied in relation to health outcomes; this body of research identifies a social gradient with those of higher socioeconomic status (SES) having better health. For example, this gradient has been well-documented in the Whitehall longitudinal studies of more than 10,000 British civil servants [1]. These studies document an inverse relationship between employment grade and the incidence of a number of health outcomes, including chronic bronchitis and coronary heart disease (CHD) mortality. They also show that many of the conventional risk factors for CHD, such as smoking, higher plasma glucose and low physical activity are more prevalent among lower grade employees; however, the social gradient is only partially explained when these potential mechanisms are accounted for [2]. Follow-up studies showed the stability of the inverse gradient between employment grade and health [3-6]. In addition, the role of income inequalities rather than simple income was emphasized, suggesting that relative income and position in the social hierarchy may be measures of inequality that are particularly relevant to health outcomes [7-9]. The literature on social inequalities in children’s health is less well developed and the social gradient has only been reported in relation to a limited number of health problems or health profiles [10]. A number of studies that have focused on children have supported the social gradient found among adults. For example, Starfield, Robertson and Riley found that social class was positively associated with health when considering minor and major physical disorders (for example, colds, infections, allergies, diabetes, epilepsy) [10]. Similarly, Emerson, Graham and Hatton showed that household income was positively associated with poor health outcomes in children on 13 out of 22 indicators (for example, children from higher incomes were less likely to suffer from headaches, psychiatric and emotional disorders), indicating the continuity of social gradient in health from childhood to adulthood [11].

### Social inequalities in mental health

Initial findings of the social gradient in children’s health were the foundation of further research concerning social gradients in mental health and adjustment. Fryers, Melzer and Jenkins conducted a systematic review of social inequalities in ‘common mental disorders’ (that is, anxiety, depression), and concluded that the majority of the studies showed these mental disorders are more prevalent in lower socioeconomic groups [12]. Similarly, a social gradient in adolescents’ emotional problems has been reported, with adolescents from the lower social class experiencing more emotional problems [13]. The authors also suggested that this relationship may be mediated by psychosocial processes that result from the widening social gradient (that is, relative inequality), for example family factors.

### Social gradients in child and adolescent antisocial behavior

Antisocial behavior among young people poses an important challenge to the United Kingdom and many other societies across the world. As well as causing great suffering for both victim and perpetrator, antisocial behavior presents a significant economic challenge. Knapp, Scott, and Davies measured both direct and indirect costs of severe antisocial behavior during childhood in the UK and found that the annual average cost per family was £15,382 (inflation corrected for 2011, approximately £19,881), with 37% of the burden taken by families [14]. A body of research indicates that antisocial behavior problems in young people have been becoming worse, at least since the 1970s. Using comparable measures, a substantial increase in adolescent antisocial behavior between 1974 and 1999 has been noted [15]. A more recent study found a small but statistically significant decrease in mean levels of conduct problems between 1999 and 2004 as reported by parents and adolescents themselves [16], though prevalence rates remained high. In the United States, adolescents’ problematic behavior rates significantly increased between 1976 and 1989 [17]. Subsequent decreases in problematic behaviors between 1989 and 1999 have been found [18], though the rates remained higher than in 1976. Taken together this literature highlights the importance of developing our understanding of antisocial behavior with the goal of informing policies to reduce the scope of the problem.

Antisocial behavior is a heterogeneous concept, including but not limited to behaviors defined as criminal. Different forms of antisocial behavior may be heterogeneous in terms of risk factors, prognosis and intervention response [19]. In the Diagnostic and Statistical Manual of Mental Disorders (DSM-IV-TR) [20] psychiatric nosology, antisocial behavior during adolescence is described within the areas of Oppositional-Defiant Disorder (ODD) and Conduct Disorder (CD). Importantly, the DSM-IV-TR enables clinicians and researchers to standardize their diagnoses so that they refer to detailed descriptions of a certain condition, including potential risk factors, developmental pathways or recovery [21]. There have been questions raised regarding whether dichotomous diagnoses should be given or whether antisocial behavior should be treated as a continuum. Such dimensional approaches have been largely supported by research demonstrating their superior predictive validity [22]. However, dichotomies remain useful as they do not seem to affect conclusions and can be easily presented in a widely understandable manner to the general public [23]. Therefore, both dichotomous and continuous measures are important approaches for measuring antisocial behavior.

ODD is characterized by conflict with adults (for example, defiance, spitefulness, loss of temper), and has a prevalence rate of approximately 3% [24]. By contrast, CD which has a prevalence of approximately 6% [24], refers to ‘a repetitive and persistent pattern of behavior in which the basic rights of others or major age-appropriate societal norms or rules are violated’ [20; p. 93] (for example, bullying, fighting and cruelty to people or animals, theft, truancy, destruction of property). The distinction between these two disorders addresses in part the heterogeneity present within antisocial behavior. However, heterogeneity remains within these diagnostic categories to some extent. For example, within the diagnosis of CD, symptoms may be meaningfully classified as involving physical aggression or not, as childhood or adolescent onset [25], or as presenting with or without callous-unemotional (CU) traits [26].

A relationship between social inequalities and broadly operationalized antisocial behavior (for example, CD, delinquency, violence, crime) has been noted in the literature [27,28]. Emerson, Graham and Hatton found that CD had significantly higher prevalence rates in the lowest income quintiles, and its prevalence gradually decreased across the income gradient [11]. Moreover, Costello, Compton, Keeler and Angold found that children from consistently poor families and those who had later moved out of poverty had significantly more symptoms of CD and ODD than never-poor children. The symptoms of children who moved out of poverty significantly decreased to the level of never-poor children after a boost to income was introduced [29]. Social inequality has been reported as strongly and positively associated with higher rates of both acquisitive and non-acquisitive crimes [30]. Taken together, these findings suggest an inverse relationship between social position and different forms of antisocial behavior, with disadvantaged children experiencing more behavioral problems [31]; yet this relationship has not been thoroughly described, particularly with reference to comparisons of different forms of antisocial behavior.

In order to understand this relationship, a few studies have addressed the mechanisms underlying the relationship between inequality and antisocial behavior. Conger and colleagues proposed a model suggesting that economic difficulties and pressure (that is, low income, debts and income loss) influence parental emotional problems, family interactions and conflict with children over money [32]. This may, in turn, lead to general hostility towards children, affecting their internalizing and externalizing problems. Such a model has been further developed using an interactionist perspective on the relationship between socioeconomic status and antisocial behavior, specifically suggesting that the relationship between socioeconomic status and child development is dynamic and changes over time [33]. Other research addressing mediators of the effect of social position on antisocial behavior suggest that socialization processes, family characteristics and child-rearing practices, including lack of warmth, harsh discipline [27] and diminished parental supervision [29,34] may play a role.

Despite the importance of the topic, the evidence on the nature of the social gradient in antisocial behavior is limited. Researchers interested in other risk processes have often treated socioeconomic status as a contextual or confounding variable in relation to antisocial behavior [35,36]; hence, many findings concerning social inequalities in antisocial behavior are spread across the literature. Therefore, a review of the evidence regarding the social gradient in antisocial behavior with the consideration of its heterogeneity and potential underlying mechanisms is needed. This will guide future enhancements of developmental models of antisocial behavior and inform policy interventions to reduce levels of antisocial behavior through minimizing the effects of social inequalities on the development of children and adolescents.

## Methods/Design

### Objectives

This systematic review aims to synthesize findings concerning the social gradient in broadly defined antisocial behavior as well as processes involved in this relationship. We will examine whether social gradients differ for subtypes of antisocial behavior. For instance, it could be hypothesized that specific CU traits shown to be particularly highly heritable [37] may have weaker associations with socioeconomic status than less heritable subtypes. Furthermore, the potential mechanisms underlying the social gradient in antisocial behavior, risk factors and mediators will be investigated in order to explore the mechanisms underlying the gradient. For example, the low parental self-efficacy, general parental dysfunction [38], poor supervision and inconsistent discipline [35] associated with children’s antisocial behavior could partly account for its relationship with socioeconomic status.

### Search strategy

**Figure 1:**
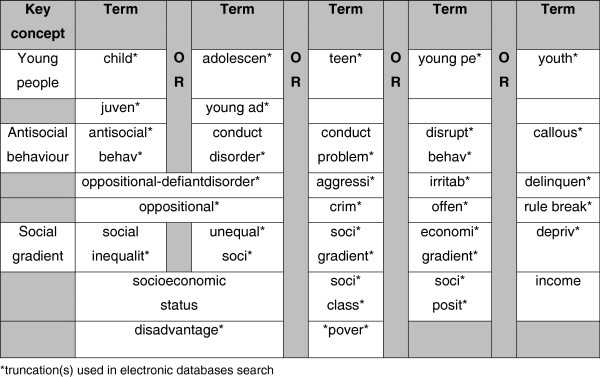
Search terms to be used in electronic databases.

The literature search will involve both manual and electronic searches, emailing experts in the field, searching grey literature and official statistics as well as backward and forward reference searching from the identified relevant papers and a manual search of key journals. Identified electronic resources will be searched using combinations of key terms (Figure 1). The search will cover the following databases: PsycInfo, Web of Knowledge, Scopus, Medline, CINAHL, Applied Social Sciences Index and Abstracts (ASSIA), Sociological Abstracts, Worldwide Political Science Abstracts, National Criminal Justice Reference Service, EconLit, System for Information on Grey Literature in Europe, UK National Statistics, and Education Resources Information Center (ERIC). Publication date restriction will be used so that only articles published from 1960 to 2011 will be retrieved.

Both CD and ODD capture a range of symptomatic behaviors from lost temper, frequent arguments, anger, and spitefulness to physical cruelty, fighting, stealing, and serious rule violations. These constructs are directly addressed in the psychiatric literature but similar constructs may be measured across different domains such as criminology, sociology and law. Consequently, a wide range of terms used by different disciplines will be incorporated in the search strategy to ensure all relevant research on antisocial behavior and social inequalities is identified. On the basis of the discussion presented earlier, the variety of antisocial behavior measures used, both categorical and dimensional, will be accepted in the review. This will allow investigation of both clinical diagnoses of disruptive behavior disorders, and also continuous scales of clinical symptoms, offending, aggression and delinquency.

Search terms, databases, date limits and initial inclusion criteria were chosen on the basis of a scoping review and on consultation of search terms used in previous reviews [39,40].

### Selection

Initial search and first stage reviewing, including assessing the relevance of articles on the basis of titles and abstracts, will be conducted by the first author only. However, when studies are found difficult to classify, corresponding decisions will be made upon discussion and agreement between the two reviewers. When retrieved, both reviewers will independently evaluate articles against explicit inclusion criteria. This process will be piloted first to ensure matching interpretation and understanding of inclusion criteria across both reviewers. Only empirical, quantitative studies of children and adolescents (<=18 years old) recruited from the general, non-clinical population, measuring both social position (for example, occupational/employment, income, or educational indicators) and broadly conceptualized antisocial behavior, that were published in English will be included in the review. A wide range of carer’s social position measures will be accepted in the review. These may include occupation, education, income and social class and they may be measured using continuous, ordinal, categorical scales, including dichotomies. A wide range of antisocial behavior measures will also be accepted, including diagnoses of CD and ODD and continuous questionnaire measures or symptoms counts for constructs such as delinquency and aggression. A detailed record of the number of studies excluded at each stage will be kept as well as the primary reason for exclusion during the second stage reviewing. The flow diagram of the selection process will be presented according to the Preferred Reporting Items for Systematic Reviews and Meta-Analyses (PRISMA) guidelines [41].

### Data extraction and quality appraisal

A standardized and pre-piloted data extraction form [See Additional file 1 will be used to extract all the relevant information for quality appraisal and synthesis. Data extraction will be undertaken by the first reviewer, although the process will be discussed and piloted by both reviewers. Subsequently, 20% of the sample will be verified by the second reviewer. All identified papers will be critically appraised independently by both reviewers. Appraisal will be guided by a checklist assessing clarity of aims and research questions, methodological quality (that is, reliability of measures), sampling techniques used, the study’s relevance to the review, inclusion of different types of antisocial behavior and analyses of mediating effects [See Additional file 2. This checklist has been developed and piloted by the first author, showing good face validity. If the psychometric properties of the instruments used are not reported, these may be taken from the other papers using the same measures. Each study will be assessed on all the quality criteria as poor, satisfactory or good scored as 0, 1, 2, respectively, which will then constitute the overall quality score of the study. This may be taken into account during synthesis as studies of high- versus low-quality may be accordingly grouped. The process of the quality appraisal will be first piloted by both reviewers. Cohen’s Kappa inter-rater reliability statistic [42] will be calculated using the quality scores assigned independently by reviewers to measure the consistency of appraisal.

### Analysis and synthesis

If the nature of the identified studies allow, meta-analysis will be conducted either on all studies or on a group of studies sufficiently homogeneous in regard to the construct measured, quality, scope, and so on. We will use META program [43] to compute effect sizes and present an N-weighted average coefficient to account for the sample size. The decision regarding applying fixed- versus random-effects models will be made at the later stages of the review process, upon assessing the heterogeneity of the included studies. If the selected studies show substantial heterogeneity, only narrative synthesis may be employed. Studies will be logically grouped into meaningful categories (for example, gradient-only studies, and those describing possible mechanisms). The basic characteristics and findings of each of the studies will be tabulated, and more weight will be given to studies assessed as ‘higher quality’ in the cross-study synthesis. If studies differing on quality scores appear to present dissimilar findings, separate conclusions may be drawn on the basis of low- versus high-quality papers, and between-study differences further examined. The same procedures may be applied to methodologically different studies, again with separate findings presented. Final synthesis will present the overall findings on the social gradient in antisocial behavior taking into account methodological variations between studies.

## Discussion

The review will synthesize findings on social gradients in different forms of antisocial behavior and potential mediating mechanisms that have been sparsely reported in the literature to date. The review will also serve as a foundation for future research that could further develop existing theories of antisocial behavior, and inform policies regarding interventions that focus on reducing social inequalities in antisocial behavior. The findings could inform the design of future research resulting in more thorough studies of the role of socioeconomic variables in behavioral problems, rather than considering them as solely contextual variables [32,33]. Variation in the social gradient effects across different forms of antisocial behavior could guide researchers to treat antisocial behavior as a multi-dimensional construct and include multiple measures to assess its different forms in their designs.

## Competing interests

The authors declare that they have no competing interests.

## Authors’ contributions

PJP and RR designed the study and developed the search strategies. PJP drafted, edited and registered the protocol. RR and CBS contributed to the draft protocol and provided feedback. All authors read and approved the final manuscript.

## Supplementary Material

Additional file 1 **Data Extraction Form.** This form will be used to extract the relevant information from each study included in the review. It covers general study information such as reference and publication type but mainly focuses on study’s design and findings, for example sample or statistics used. It will serve as guidance for the reviewers and the form may be revised in due course. Click here for file

Additional file 2 **Quality Appraisal Checklist.** This checklist contains all the quality aspects mentioned in the article (for example, methodological quality and analyses applied), which will be critically appraised and scored as previously described. Click here for file

## References

[B1] Marmot M Brunner E Cohort profile: The Whitehall II study Int J Epidemiol 2005 34 251 256 10.1093/ije/dyh372 15576467

[B2] Marmot MG Rose G Shipley M Hamilton PJS Employment grade and coronary heart-disease in British civil-servants J Epidemiol Commun Health 1978 32 244 249 10.1136/jech.32.4.244 PMC1060958 744814

[B3] Marmot MG Shipley MJ Rose G Inequalities in death – specific explanations of a general pattern Lancet 1984 1 1003 1006 6143919 10.1016/s0140-6736(84)92337-7

[B4] Davey Smith G Shipley MJ Rose G Magnitude and causes of socioeconomic differentials in mortality: further evidence from the Whitehall Study J Epidemiol Commun Health 1990 44 265 270 10.1136/jech.44.4.265 PMC1060667 2277246

[B5] Marmot MG Shipley MJ Do socioeconomic differences in mortality persist after retirement? 25 year follow up of civil servants from the first Whitehall study Brit Med J 1996 313 1177 1180 10.1136/bmj.313.7066.1177 8916748 PMC2352486

[B6] van Rossum CTM Shipley MJ van de Mheen H Grobbee DE Marmot MG Employment grade differences in cause specific mortality. A 25 year follow up of civil servants from the first Whitehall study J Epidemiol Commun Health 2000 54 178 184 10.1136/jech.54.3.178 PMC1731642 10746111

[B7] Wilkinson RG Socioeconomic determinants of health - health inequalities: relative or absolute material standards? Brit Med J 1997 314 591 595 10.1136/bmj.314.7080.591 9055723 PMC2126067

[B8] Wilkinson RG Comment: income, inequality, and social cohesion Am J Public Health 1997 87 1504 1506 10.2105/AJPH.87.9.1504 9314804 PMC1380977

[B9] Marmot M The influence of income on health: views of an epidemiologist Health Aff (Millwood) 2002 21 31 46 11900185 10.1377/hlthaff.21.2.31

[B10] Starfield B Robertson J Riley AW Social class gradients and health in childhood Ambul Pediatr 2002 2 238 246 10.1367/1539-4409(2002)002<0238:SCGAHI>2.0.CO;2 12135396

[B11] Emerson E Graham H Hatton C Household income and health status in children and adolescents in Britain Eur J Public Health 2006 16 354 360 10.1093/eurpub/cki200 16207724

[B12] Fryers T Melzer D Jenkins R Social inequalities and the common mental disorders - a systematic review of the evidence Soc Psych Psych Epid 2003 38 229 237 10.1007/s00127-003-0627-2 12719837

[B13] Langton EG Collishaw S Goodman R Pickles A Maughan B An emerging income differential for adolescent emotional problems J Child Psychol Psychiatry 2011 52 1081 1088 10.1111/j.1469-7610.2011.02447.x 21815893

[B14] Knapp M Scott S Davies J The cost of antisocial behaviour in younger children Clin Child Psychol Psychiatry 1999 4 457 473 10.1177/1359104599004004003

[B15] Collishaw S Maughan B Goodman R Pickles A Time trends in adolescent mental health J Child Psychol Psychiatry 2004 45 1350 1362 10.1111/j.1469-7610.2004.00335.x 15482496

[B16] Maughan B Collishaw S Meltzer H Goodman R Recent trends in UK child and adolescent mental health Soc Psych Psych Epid 2008 43 305 310 10.1007/s00127-008-0310-8 18250944

[B17] Achenbach TM Dumenci L Rescorla LA Are American children's problems still getting worse? A 23-year comparison J Abnorm Child Psych 2003 31 1 11 10.1023/A:1021700430364 12597695

[B18] Achenbach TM Dumenci L Rescorla LA Ten-year comparisons of problems and competencies for national samples of youth: self, parent, and teacher reports J Emot Behav Disord 2002 10 194 203 10.1177/10634266020100040101

[B19] Burke JD Loeber R Birmaher B Oppositional defiant disorder and conduct disorder: a review of the past 10 years, part II J Am Acad Child Psychiatry 2002 41 1275 1293 10.1097/00004583-200211000-00009 12410070

[B20] American Psychiatric Association Diagnostic and Statistical Manual of Mental Disorders.Text Revision (TR) 2000 4 Washington, DC: American Psychiatric Association

[B21] Goodman R Scott S Child Psychiatry 2005 2 Oxford: Blackwell Publishing

[B22] Fergusson DM Horwood J Predictive validity of categorically and dimensionally scored measures of disruptive childhood behaviors J Am Acad Child Adolesc Psychiatry 1995 34 477 485 10.1097/00004583-199504000-00015 7751262

[B23] Farrington DP Loeber R Some benefits of dichotomization in psychiatric and criminological research Crim Behav Ment Health 2000 10 100 122 10.1002/cbm.349

[B24] Green H McGinnity A Meltzer H Ford T Goodman R Mental Health of Children and Young People in Great Britain. Summary Report 2004 London: Her Majesty's Stationary Office 2005

[B25] Moffitt TE Adolescence-limited and life-course-persistent antisocial behavior: a developmental taxonomy Psychol Rev 1993 100 674 701 8255953

[B26] Scheepers FE Buitelaar JK Matthys W Conduct disorder and the specifier callous and unemotional traits in the DSM-5 Eur Child Adoles Psy 2011 20 89 93 10.1007/s00787-010-0149-x PMC3038229 21140182

[B27] Dodge KA Pettit GS Bates JE Socialization mediators of the relation between socioeconomic-status and child conduct problems Child Dev 1994 65 649 665 10.2307/1131407 8013245

[B28] Wilkinson R Why is violence more common where inequality is greater? Ann NY Acad Sci 2004 1036 1 12 15817728 10.1196/annals.1330.001

[B29] Costello EJ Compton SN Keeler G Angold A Relationships between poverty and psychopathology - A natural experiment JAMA 2003 290 2023 2029 10.1001/jama.290.15.2023 14559956

[B30] Whitworth A Inequality and crime across England: a multilevel modelling approach Soc Policy Society 2012 11 27 40 10.1017/S1474746411000388

[B31] McLeod JD Shanahan MJ Trajectories of poverty and children's mental health J Health Soc Behav 1996 37 207 220 10.2307/2137292 8898493

[B32] Conger RD Ge X Elder GH Jr Lorenz FO Simons RL Economic stress, coercive family process, and developmental problems of adolescents Child Dev 1994 65 541 561 10.2307/1131401 8013239

[B33] Conger RD Donnellan MB An interactionist perspective on the socioeconomic context of human development Annu Rev Psychol 2007 58 175 199 10.1146/annurev.psych.58.110405.085551 16903807

[B34] Lahey BB Waldman ID McBurnett K Annotation: the development of antisocial behavior: an integrative causal model J Child Psychol Psyc 1999 40 669 682 10.1111/1469-7610.00484 10433402

[B35] Frick PJ Lahey BB Loeber R Stouthamerloeber M Christ MAG Hanson K Familial risk factors to oppositional defiant disorder and conduct disorder: parental psychopathology and maternal parenting J Consult Clin Psych 1992 60 49 55 10.1037//0022-006x.60.1.49 1556285

[B36] Pardini DA Lochman JE Powell N The development of callous-unemotional traits and antisocial behavior in children: are there shared and/or unique predictors? J Clin Child Psychol 2007 36 319 333 10.1080/15374410701444215 17658977

[B37] Viding E Blair RJR Moffitt TE Plomin R Evidence for substantial genetic risk for psychopathy in 7-year-olds J Child Psychol Psychiatry 2005 46 592 597 10.1111/j.1469-7610.2004.00393.x 15877765

[B38] Kolko DJ Dorn LD Bukstein O Burke JD Clinically referred odd children with or without cd and healthy controls: comparisons across contextual domains J Child Fam Stud 2008 17 714 734 10.1007/s10826-007-9186-6

[B39] Attree P Childhood Disadvantage and Health Inequalities: A Systematic Review of the Qualitative Evidence 2004 Lancaster: Lancaster University

[B40] Pollitt R Rose K Kaufman J Evaluating the evidence for models of life course socioeconomic factors and cardiovascular outcomes: a systematic review BMC Publ Health 2005 5 7 10.1186/1471-2458-5-7 PMC548689 15661071

[B41] Moher D Liberati A Tetzlaff J Altman DG Preferred reporting items for systematic reviews and meta-analyses: the PRISMA statement PLoS Med 2009 6 e1000097 10.1371/journal.pmed.1000097 19621072 PMC2707599

[B42] Cohen J A coefficient of agreement for nominal scales Educ Psychol Meas 1960 20 37 46 10.1177/001316446002000104

[B43] Schwarzer R Meta: Programs for Secondary Data nalysis 1988 Berlin: University of Berlin

