# Supplementary material for: Social gradients in child and adolescent antisocial behavior: a systematic review protocol
Source: Syst Rev. 2012 Aug 23;1:38. doi: 10.1186/2046-4053-1-38 (PMC3485181; doi:10.1186/2046-4053-1-38)
Supplement: Additional file 1 — Data Extraction Form. This form will be used to extract the relevant information from each study included in the review. It covers general study information such as reference and publication type but mainly focuses on study’s design and findings, for example sample or statistics used. It will serve as guidance for the reviewers and the form may be revised in due course. [file 2046-4053-1-38-S1.pdf]

Data Extraction Form

Author(s)		
Year		
Publication type		
Full reference		
Source		
Objective(s)		
Design		
Country		
Quality Status		
Data Source (e.g. survey)		
Social gradient measure(s)		
Antisocial behaviour measure(s)		
Sample	Size	
	Sex, Ethnicity	
	Age range	
	Response rate	
Results	Social gradient in antisocial behaviour?	
	Significance	
	direct vs. indirect	
	Moderators	
	Mediators	
	Mechanisms	
Comments		
