# Supplementary material for: Social gradients in child and adolescent antisocial behavior: a systematic review protocol
Source: Syst Rev. 2012 Aug 23;1:38. doi: 10.1186/2046-4053-1-38 (PMC3485181; doi:10.1186/2046-4053-1-38)
Supplement: Additional file 2 — Quality Appraisal Checklist. This checklist contains all the quality aspects mentioned in the article (for example, methodological quality and analyses applied), which will be critically appraised and scored as previously described. [file 2046-4053-1-38-S2.pdf]

Quality Appraisal

Scale (max. 20 points)

0 - poor

1 - satisfactory

2 - good

No.	Criteria		Grade	Comments
**1.**	Clearly described aims, research questions and focus of a study[1].			
**2.**	Study relevance to the review[2].			
**3.**	General methodological quality and appropriateness of the design.			
**4.**	Sample:	Accurately and sufficiently described (i.e., detailed demographics).		
		Size and external validity; is it representative? (e.g., responders versus non-responders, weighting, sampling)		
**5.**	Reliable, valid and well described measures of all the variables.			
**6.**	Measures of different forms of antisocial behaviour[3].			
**7.**	Analyses of potential mediating and/or moderating effects.			
**8.**	Sufficient and relevant to the review analyses coherently presented.			
**9.**	Valid conclusions and its overall contribution to the review (i.e. addressing the research questions).			

Overall Quality Status:

(0-6 → poor; 7-13 → average/acceptable; 14-20 → good)

[1] Each relevant study reported in a paper to be critically appraised.

[2] Simply reported a relationship between SES and broadly defined antisocial behaviour [poor]; investigation of the relationship and/or inclusion of different forms of antisocial behaviour [average]; thorough investigation of the relationship, including mediating effects etc. [good]

[3] Different types of crimes/offences will be considered as different forms of antisocial behaviour.
